# Larval Habitats Characteristics of Mosquitoes (Diptera: Culicidae) in North-East of Iran

**Published:** 2017-05-27

**Authors:** Aioub Sofizadeh, Seyed Hassan Moosa-Kazemi, Hossein Dehghan

**Affiliations:** 1 Infectious Diseases Research Center, Golestan University of Medical Sciences, Gorgan, Iran; 2 Department of Medical Entomology and Vector Control, School of Public Health, Tehran University of Medical Sciences, Tehran, Iran

**Keywords:** Larval habitats, Mosquitoes, Iran

## Abstract

**Background:**

There are unorganized, published documents about the ecology of mosquitoes (Diptera: Culicidae) in northeastern part of Iran. The purpose of this study was to determine the distribution and characteristics of larval habitats of Culicidae in Kalaleh County.

**Methods:**

Larvae were collected using dipping method and adults by human landing catch technique during April–October, 2012. Larval habitat characteristics were recorded such as vegetation status, and sunlight, water situation. Lacto-phenol and de Faure’s media were used for conserving and mounting samples. Data were analyzed using SPSS statistical software, version 11.5.

**Results:**

Out of the 395 larvae collected, 332 were adult mosquitoes comprising; *Culiseta*, *Culex*, *Anopheles* and *Ochlerotatus* genera and 14 species including *An. superpictus*, *An. maculipennis* s.l., *An. hyrcanus*, *An. psudopictus*, *An. claviger*, *Culex pipiens*, *Cx. theileri*, *Cx. perexiguus*, *Culiseta longiareolata*, *Cs. subochrea*, *Ochlerotatus caspius*, *Oc. echinus* and *Oc. geniculatus*. *Culex pipiens* larvae were predominant (27.6%) and *Cs. subochrea* (1%) was found as the lowest species in terms of number. In the adult form, *Cx. pipiens* (28.9%) was predominant whereas, *Cs. subochrea* and *Cx. perexiguus* were reported to have had the lowest frequency*.*

**Conclusion:**

The larvae of *An. superpictus* and *An. maculipennis* species as the main vectors of malaria in north of Iran were reported in permanent habitats with clear water and vegetation, full and partial sunlight situations and muddy as well as sandy substrates that are important in larvicide application programs. Exclusive studies are necessary to diagnose *An. maculipennis* species complex using molecular and morphological analysis in the future.

## Introduction

Culicidae family is one of the largest and most medically important families of Diptera. By now, 64 species and 3 subspecies have been identified in seven genera and 16 subgenera in Iran (Azari-Hamidian 2007a). Habitats of the mosquito larval stages affect the distribution pattern of adult stages. Mosquito habitats are classified as natural or artificial, permanent or temporary. Indeed, larval habitats are considered as specific for each mosquito species. Moreover, studies on mosquito larval habitats could be useful for vector control programs (Bruce-Chawat 1980).

There are scattered studies on bionomics and ecology of mosquitoes in northeast of Iran. Macan (1950) mentioned some ecological aspects of *Anopheles* species in the near East of Iran. Dow (1953) reported some characteristics of larval habitats of six *Culex* species. Larval habitats of *Cx. pipiens* were previously studied in Tehran Province (Golestani 1967). Lotfi (1970, 1973, and 1976) studied temperature and pH of larval habitats of mosquito larvae in Iran. The characteristics of larval habitats of mosquitoes were subsequently reported in Minab area, south of Iran (Yaghoobi-Ershadi et al. 1986). The distribution and characteristics of larval habitats of mosquitoes in Iran were studied by Zaim in 1987. The ecology and fauna of mosquitoes were reported in Esfahan Province (Mousa-Kazemi et al. 2000a). Azari-Hamidian (2005, 2006, 2007b, 2011) and Azari-Hamidian et al. (2011) reported diversity and larval habitats of mosquitoes in the north of Iran. Besides, physical and chemical factors affecting larval habitats of *Anopheles* species were studied in southeast of Iran (Ghanbari et al. 2005). Some studies about the ecology and fauna of mosquitoes were reported in Neka County, northern part of Iran (Nikookar et al. 2015). Ecology and morphological characteristics of mosquitoes were reported in Yazd City, central Iran (Dehghan et al. 2010). Larval habitats and biodiversity of anopheline mosquitoes and some environmental characteristics were studied in southern Iran (Hanafi-Bojd et al. 2012, Soleimani-Ahmadi et al. 2013).

There are scattered information about fauna and ecological characteristics of mosquitoes in Golestan Province. By now, 10 *Anopheles* and 14 Culicinae species were identified using morphological characters and the surface patterns of eggs. Earlier studies had been conducted in northeastern part of Iran including Mazandaran and north Khorasan Provinces (Gutsevich 1943, Zolotarev 1945, Dow 1953, Zaim 1987, Sedaghat et al. 2003, Nikookar et al. 2015).

Mosquito-borne diseases including malaria, arboviral diseases and filariasis are the most common arthropod borne diseases in the world (Gubler 1998). Presently, malaria is one of the most important problems in Iran. Golestan Province was one of the malaria foci in Iran but there are no imported cases in the province. Recently, number of endemic foci of malaria has been identified in different neighboring countries of Iran including Afghanistan, Pakistan and Tukmenistan and potential vectors are widely dispersed. However, a rapid spread of the diseases is likely to occur due to the lack of vector control programs (Ministry of Health and Medical Education, 2012).

Epidemics occur in Turkmenistan, a neighboring country of Iran, climate change and the imported cases are considered as the most reasons for the increase in diseases from 2003–2004 (Ministry of Health and Medical Education, 2012). Kalaleh County is located in the north-east of Iran. Because of favorable weather which supports the breeding of mosquitoes, risk of malaria transmission, immigration and lack of malaria control, it is important to obtain adequate information in the field of malaria epidemiology in order to optimize the implementation of fundamental research programs. In addition, study on the ecology of malaria vectors in this area will help obtain better management of vector control and proper approach to malaria control programs.

Therefore, this study was carried out to determine some ecological aspects of the Culicidae species and characteristics of their habitats in Kalaleh County, Golestan Province, northeast of Iran.

## Materials and Methods

### Study area

**Fig. 1.**
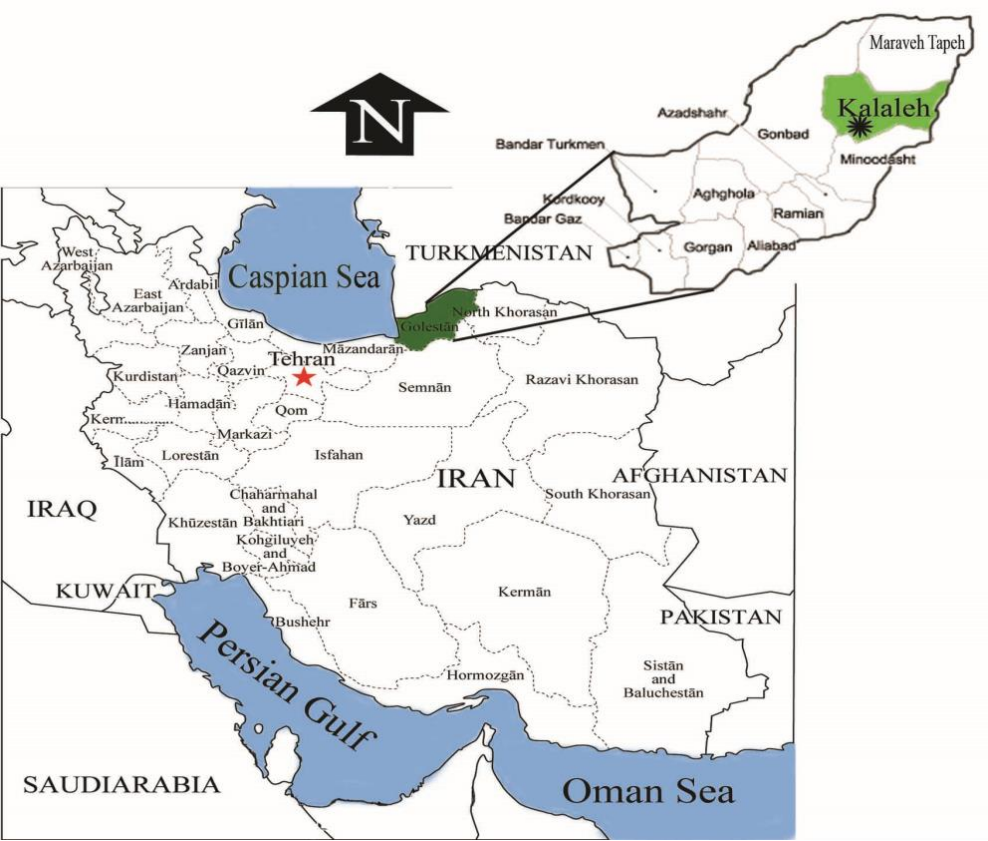
The study area of Kalaleh County, Golestan Province, North of Iran

A cross-sectional study was carried out in Kalaleh County, Golestan Province, northern Iran from April to October 2012. This study took place in seven randomly selected rural villages of the County (37° 70 ′N 55°81′ E). The samples were collected in plain, slope and mountainous areas. The province is bounded by Caspian Sea and Mazandaran Province in the West, Semnan Province in the South, North Khorasan Province in the East and Turkmenistan Country in the North (Fig. 1). Most parts of Golestan Province are plain and more than 2/3 of the plain areas have arid and semiarid climates and 1/3 of others have a mild climate. This County has 4962km and a population of 153261 people and is located in northeast of Golestan Province. The main agricultural products are *Alfa alfa*, water melon and cotton. Maximum and minimum of precipitation were recorded as 40.8 and −0.02 respectively and mean annual relative humidity was recorded as 67%. The total annual rainfall was 772mm, the minimum in August and maximum in February.

### Mosquito sampling

Sampling was carried out using dipping method for collecting mosquito larvae and night catch for adult collection. Larval sampling method was carried out using standard dipper of 350ml. Each habitat was sampled in different parts of the larval habitats for 10 times. In order to clarify the samples collected, they were conserved and transported to the laboratory in vials containing lacto-phenol solution. The vials were labeled based on sample’s date code and their associated habitats. Features such as larval habitat status (permanent or temporary, stagnant or slow-running water), vegetation type, substrate type, habitat types and position of the sunlight were recorded on special forms. The night catch method was carried out using suction tubes from animal baited traps. Animal baited collection was conducted from 18.00 PM to 03.00 AM monthly in fixed animal shelters randomly placed in each village. Sample containers were protected from light and heat and were transferred to the Laboratory of School of Public Health, Tehran University of Medical Sciences, where the authors identified the specimens using the taxonomic keys of Shahgudian 1960, Zaim and Cranston 1986, Harbach 1985, and Azari-Hamidian and Harbach 2009. The mosquito name abbreviations were cited based on Reinert (2009).

## Results

Overall, 395 larvae and 332 adult mosquitoes in 4 genera and 14 species in seven sampling places were found in this area. Four species of *Culex*, five species of *Anopheles*, two species of *Culiseta* and three species of *Ochlerotatus* were identified. The species of mosquito larvae which were reported included: *An. superpictus* Grassi, *An. maculipennis* s.l, *An. hyrcanus* (Pallas), *An. psudopictus*, *An. claviger* (Meigen), *Cx. pipiens* Linnaeus, *Cx. theileri* Theobald, *Cx. hortensis* Ficalbi, *Cx. perexiguus* Theobald, *Cs. longiareolata* (Macquart), *Cs. subochrea* (Edwards), *Oc. caspius* s.l (Pallas), *Oc. echinus* (Edwards) and *Oc. geniculatus* (Olivier). All the species were found in adult forms except *Cx. perexiguus*.

*Culex pipiens* and *Cs. longiareolata* were the dominant species reported. The larvae and adult species of *Cx. theileri*, *Cx. pipiens*, *Oc. caspius* and *Oc. echinus* in all larval habitats were collected and presented in Table 1 and 2. The association of *Cx. pipiens* larvae with the other Culicidae species was more than the other species (Table 3).

**Table 1.** Frequency of mosquito larvae which were collected by dipping method in Kalaleh County, 2012

**Places**	**Kheder -Olia jungle**	**Ghoshe saver jungle**	**Beili jungle**	**Gorgandoz**	**Barbar ghaleh**	**Sade chamran**	**Gharanki Jangal**	**total**	**Percentage**

**Species**									
***An. claviger***	1	0	1	4	2	5	1	14	3.5
***An. superpictus***	0	1	0	7	3	1	1	13	3.3
***An. maculipennis* s.l**	1	2	4	1	2	5	4	19	4.8
***An. psudopictus***	1	1	2	4	0	2	5	15	4
***An. hyrcanus***	2	4	2	6	1	0	5	20	5.1
***Cs. longiareolata***	4	12	11	10	12	11	11	71	18.2
***Cs. subochrea***	0	1	0	0	1	0	2	4	1
***Cx. theileri***	10	10	8	4	2	4	1	39	9.9
***Cx. hortensis***	2	1	3	4	3	2	5	20	5.1
***Cx. perexiguus***	1	1	4	1	4	1	5	17	4.3
***Cx. pipiens***	15	14	24	15	14	14	13	109	27.6
***Oc. caspius***	6	4	6	5	6	3	1	31	7.8
***Oc. echinus***	2	1	3	1	2	4	2	15	3.8
***Oc. geniculatus***	1	1	1	1	0	1	1	6	1.5

**Total**	46	53	69	63	52	53	57	393	100

**Table 2.** Frequency of adult mosquitoes which were collected by night catch method from animal baited traps in Kalaleh County, 2012

**Places**	**Kheder Olia jungle**	**Ghoshesaver jungle**	**Ghoshechashme jungle**	**Beili jungle**	**Parpari jungle**	**Aghsou jungle**	**Azizabad**	**total**	**Percentage**

**Species**									
***An. claviger***	0	0	1	2	5	1	3	12	3.6
***An. superpictus***	5	1	0	3	1	5	2	17	5.1
***An. maculipennis* s.l**	8	2	4	2	5	1	1	23	7
***An. psudopictus***	11	1	2	0	2	3	1	20	6
***An. hyrcanus***	12	4	2	1	0	3	3	25	7.6
***Cs. longiareolata***	4	2	3	2	1	0	4	16	4.8
***Cs. subochrea***	0	0	0	0	1	0	1	2	0.6
***Cx. theileri***	10	10	11	10	10	4	10	65	19.5
***Cx. hortensis***	0	1	0	0	1	0	0	2	0.6
***Cx. pipiens***	15	14	12	14	14	12	15	96	28.9
***Oc. caspius***	6	4	2	6	3	4	5	30	9
***Oc. echinus***	2	1	2	2	4	1	1	13	3.9
***Oc. geniculatus***	1	1	2	2	1	3	1	11	3.3

**Total**	74	41	41	44	48	37	47	332	100

**Table 3.** Association of mosquito larvae collected in Kalaleh County, 2012

**Species**	**No of larvae habitates**	***An. claviger***	***An. superpictus***	***An. maculipennis s***	***An. psudopictus***	***An. hyrcanus***	***Cs. longiareolata***	***Cs. subochrea***	***Cx. theileri***	***Cx. hortensis***	***Cx. perexiguus***	***Cx. pipiens***	***Oc. caspius***	***Oc. echinus***	***Oc. geniculatus***
***An.claviger***	5	✷	1	3	2	5	1	1	2	3	1	1	1	2	1
***An. superpictus***	5	1	✷	2	3	1	5	1	4	2	6	3	2	1	5
***An.maculipennis sl***	8	2	4	✷	2	5	1	4	3	6	2	1	5	2	6
***An. psudopictus***	11	1	2	1	✷	2	3	5	6	1	2	1	4	2	5
***An. hyrcanus***	12	4	2	3	1	✷	3	5	5	1	1	2	2	1	4
***Cs. longiareolata***	14	12	13	14	12	11	✷	11	12	14	11	9	8	7	9
***Cs. subochrea***	5	2	3	5	4	1	2	✷	1	2	5	4	3	2	1
***Cx. theileri***	15	12	14	15	12	13	14	12	✷	11	12	9	8	6	8
***Cx. hortensis***	5	1	2	4	3	2	1	5	1	✷	2	4	5	3	6
***Cx. perexiguus***	5	1	2	1	4	1	2	5	1	4	✷	4	5	3	4
***Cx. pipiens***	15	14	12	15	14	14	12	13	11	14	12	✷	12	14	15
***Oc. caspius***	6	4	2	5	6	3	4	1	5	2	5	2	✷	4	5
***Oc. echinus***	4	1	2	1	2	4	1	2	3	1	2	3	1	✷	3
***Oc. geniculatus***	3	1	2	1	2	1	3	1	2	1	3	1	2	1	✷

Larval habitats of some mosquito species were diverse. *Anopheles claviger* and *Oc. geniculatus* larvae were collected only in permanent larval habitats (Table 4). *An. claviger*, *An. superpictus*, *An. hyrcanus*, *Oc. geniculatus* larvae were found in the larval habitats without vegetation, whereas *Cs. longiareolata* and *Cs. subochrea* were collected from habitats with vegetation (Table 4). Most larval habitats were found with substrate of mud and sand bottom and fewer larvae were collected in rocks and cement substrates. Besides, total number of samples of *Anopheles* species was collected in fresh water (Table 4).

**Table 4.** Larval habitat characteristics of mosquitoes collected in Kalaleh County, 2012

**Larval habitat**	***An. claviger***	***An. superpictus***	***An. maculipennis sl***	***An. psudopictus***	***An. hyrcanus***	***Cs. longiareolata***	***Cs. subochrea***	***Cx. theileri***	***Cx. hortensis***	***Cx. perexiguus***	***Cx. pipiens***	***Oc. caspius***	***Oc. echinus***	***Oc. geniculatus***
**Habitat**														
**Permanent**	100	92.8	92.7	64.3	95.5	77.2	21	73	65.3	36.1	93.8	93	89	100
**Temporary**	0	7.2	7.3	35.7	4.5	22.8	79	27	34.7	63.9	6.2	7	11	0
**Slow-running water**	8	55.5	85	63	12	32	45	64	2.3	9	65.5	61	69	100
**Stagnant water**	92	45.5	15	37	88	68	55	36	97.7	91	35.5	39	31	0
**Vegetation**														
**With**	0	0	95.3	89	0	100	100	55	36	59	69.7	59	94	0
**Without**	100	100	4.7	11	100	0	0	45	64	41	29.3	41	6	100
**Substrate**														
**Mud**	100	80	14	39	79	69	65	45	65	73	31.3	96	89	100
**Sand**	0	20	86	61	21	21	35	35	25	27	47.7	3	11	0
**Rock and Cement**	0	0	0	0	0	10	0	20	10	0	21	1	0	0
**Water Situation**														
**Turbid**	0	0	0	0	0	65	78	64	49	61	81	79	0	55
**Clear**	100	100	100	100	100	35	22	36	51	39	19	21	100	45
**Sunlight situation**														
**Full sunlight**	0	0	94.5	79	21.8	89	24	56	61	59	63	69	69	56
**Partial sunlight**	65	65	5.5	21	41	11	76	44	39	41	37	31	31	44
**Shaded**	35	35	0	0	37.2	0	0	0	0	0	0	0	0	0
**Habitat Kind**														
**Natural**	100	55.9	55.8	35.5	100	65	82	87	71	89	100	36	74	69
**Artificial**	0	44.1	44.2	64.5	0	35	18	13	29	11	0	64	26	31

## Discussion

In our study, a total of 395 larvae and 332 adults were found in 4 genera and 14 species. The mosquito species that were identified included, *An. claviger*, *An. hyrcanus*, *An. maculipennis* s.l, *An. psudopictus*, *An. superpictus*, *Cx. hortensis*, *Cx. perexiguus*, *Cx. pipiens*, *Cx. theileri*, *Cs. longiareolata*, *Cs. subochrea*, *Oc. caspius*, *Oc. echinus* and *Oc. geniculatus*.

The checklist of Culicidae has been prepared and reported in Mazandaran, Golestan and North-Khorasan Provinces (Dow 1953, Zaim 1987, Sedaghat et al. 2003, Sedaghat and Harbach 2005, Azari-Hamidian et al. 2011, Nikookar et al. 2015). The mosquito species which were recorded in this area were discovered by other authors who used PCR technique and those that were not identified or reported in our study are shown by asterisk (*) as follows:

*Anophles claviger* Meigen, *An. hyrcanus* Pallas, *An. maculipennis* Meigen, *An. melanoon* Hackett*, *An. persiensis* Linton, Sedaghat and Harbach*, *An. plumbeus* Stephens*, *An. pulcherrimus* Theobald*, *An. pseudopictus* Grassi, *An. sacharovi* Favre*, *An. superpictus* Grassi, *Aedes vexans* Meigen*, *Culex hortensis* Ficalbi, *Cx. mimeticus* Noe*, *Cx. perexiguus* Theobald, *Cx. pipiens* Linnaeus, *Cx. theileri* Theobald, *Cx. tritaeniorhynchus* Giles*, *Cx. modestus* Ficalbi*, *Culiseta annulata* Schrank*, *Cs. longiareolata* Macquart, *Cs. subochrea* Edwards, *Ochlerotatus caspius* s.l. Pallas, *Oc. echinus* Edwards, *Oc. geniculatus* Olivier, Oc. pulcritarsis* Rondani, *Uranotaenia unguiculata* Edwards*.

Dow (1953) mentioned *Anopheles* mosquito fauna in Gorgan (Aliabad and Ramian) including: *An. hyrcanus* var. *pseudopictus* (in the now *An.pseudopictus*), *An. pulcherrimus*, *An. superpictus*, and *An. maculipennis* group (*An. maculipennis*, *An*. *melanoon* subspecies *subalpinus* (in the now subspecies of “*subalpinus*” is synonym of “*melanoon*”) (in and *An*. *sacharovi*). *Anopheles pulcherrimus* was reported from Ali-Abad of Golestan Province by Dow in 1953. This species was reported in North-Khorasan (Azari-Hamidian et al. 2011), Moreover, the occurrence of this species in Golestan Province needs more considerations for future studies. Sedaghat et al. (2003) reported the occurrences of *An. maculipennis*, *An. sacharovi*, *An. persiensis* based on molecular identification and ITS2 sequences in Mazandaran Province which was bordered with Golestan. Sedaghat and Harbach (2005) confirmed the presence of *An. melanoon*, *An. persiensis* and *An*. *pseudopictus* species in Mazandaran Province.

Zaim (1987) reported 12 Culicinae species in Mazandaran including: *Ae. vexans*, *Oc. geniculatus*, *Oc. pulcritarsis*, *Oc. echinus*, *Cx. hortensis*, *Cx. mimeticus*, *Cx. perexiguus*, *Cx. pipiens*, *Cx. theileri*, *Cx. tritaeniorhynchus*, *Culiseta longiareolata*, *Cs. subochrea*. Nikookar et al. (2015) reported nine species of mosquito including: *An. claviger*, *An. maculipennis*, An*. plumbeus*, *An. superpictus*, *Cs. annulata*, *Cs. longiareolata*, *Cx. mimeticus*, *Cx. pipiens*, and *Oc. geniculatus* in Neka County, Mazandaran Province. Azari-Hamidian et al. (2011) reported fourteen species of mosquito representing five genera in North-Khorasan Province including: *An. claviger*, *An. maculipennis*, *An. superpictus*, *An. pulcherrimus*, *Cx. hortensis*, *Cx. mimeticus*, *Cx. modestus*, *Cx. perexiguus*, *Cx. pipiens*, *Cx. theileri*, *Cx. tritaeniorhynchus*, *Cs. longiareolata*, *Oc. caspius* and *Ur. unguiculata*

In our research, *An. claviger* was collected in permanent and stagnant habitats with muddy substrate, clear water, without vegetation. This species was only collected from natural habitats. In parallel, larval habitats of this species were reported in spring pools with partial sunlight, slow running water and shaded streams in Iraq and western Iran (Macan 1950). Other larval habitats of this species were expressed as small shallow and shaded stream, with vegetation in Maragheh area in northwestern part of Iran (Dow 1953). Nikookar et al. (2015) had found the larvae of *An. claviger* in permanent and stagnant water environments with vegetation and clay and stone substrate. In parallel, Macan (1950) had found *An. claviger* larvae in semi sunlight springs, and slow running pools of water in Iraq and western Iran. Dow (1953) reported the larval habitat of this species in shallow and small pools with little vegetation.

In this present study, *An. maculipennis* larvae were mainly collected from permanent and slow running water environments with vegetation. The other characteristics of larval habitat of the species were found as clear water, sunlight situations, and habitats with sandy substrate. The presence of *An. maculipennis* larvae was reported in permanent, transparent, semi-shady natural larval habitats with vegetation and cement or stone substrate (Nikookar et al. 2015). In parallel, the larva of this species was found in habitats with gravel substrate, sunny springs, and pools with stagnant water (Azari-Hamidian 2007b, Azari-Hamidian et al. 2011). At least 12 palearctic members of *An. maculipennis* complex were reported including *An. atroparvus*, *An. beklemishevi*, *An. labranchiae*, *An. maculipennis*, *An. martinius*, *An. melanoon*, *An. messeae*, *An. sacharovi*, *An. persiensis*, *An. daciae*, *An. lewisi* and *An. Artemievi* (White 1978, Ribeiro et al. 1988, Linton et al. 2002, [Bibr B46][Bibr B47] Djadid et al. 2007). Dow (1953) had reported the occurrence of *An. subalpinus* (in the now “*subalpinus*” is synonym of “*melanoon*”) in Sari, Babolsar, Mazandaran Province.

Saebi (1987) also cited the occurrence of *An. messeae* and *An. melanon* from Guilan Province, and *An. sacharovi* and *An. hyrcanus* in Golestan Province. This species has been identified in Guilan Province (Azari-Hamidian et al. 2004), Mazandaran and Golestan Provinces (Zaim et al. 1986). *Anopheles maculipennis s.l.* associated with *An. hyrcanus*, *An. claviger* from Mazandaran Province previously (Nikookar et al. 2015). *An. sacharovi was* cited in Mazandaran and Golestan Provinces (Sedaghat et al. 2003). Presently, five members of *An. maculipennis* complex have been reported. *Anopheles maculipennis* and *An. sacharovi* were identified based on the characteristics of eggs, larvae and adults as well as through the PCR technique, *An. messeae*, *An. persiensis* and *An. melanoon* were identified based on pattern of eggs surface and PCR technique (Sedaghat and Harbach 2005). *Anopheles maculipennis* was reported more in rice fields, while *An. sacharovi* was found more in mountainous areas (Mousa-Kazemi et al. 2000, Sedaghat et al. 2003). Although, it is difficult to find the difference between *An. maculipennis* and *An. sacharovi* species in larval stages, but in our research *An. maculipennis* species was identified based on the Azari-Hamidian and Harbach (2009)’s systematic key.

*Anopheles superpictus* was reported as one of the main malaria vectors and salivary infection was found as ranging from 0.65 to 4.6% (Manuchehri et al. 2003). This species with *An. maculipennis* was considered as the malaria vector during the outbreak of the diseases which had occurred in Azerbaijan at the borderline of the country, Armenia, and Turkey countries in 1990. However, after the independence of the southern republics of the former Soviet Union, Iran was threatened by imported malaria cases (Oshaghi et al. 2011). In present study, *An. superpictus* was collected in natural habitats. The characteristics of larval habitat of this species were mainly in permanent water without vegetation, clear water, semi-sunlight and shaded habitats with muddy substrate. Zolotarev (1945), Dow (1953) and Nikookar et al. (2015) have reported the occurrence of this species in Mazandaran Province. *Anophles superpictus* larvae was found in permanent, stagnant, with muddy substrate, transparent water, semi-shady, natural with vegetation habitats in Neka county, northern Iran (Nikookar et al. 2015). Moreover, Azari-Hamidian et al. (2011) have stated its presence in stagnant, transient, muddy substrate, full sunlight water with vegetation in natural habitats in Guilan Province, northern Iran. Further support for our results comes from some previous studies carried out in Kermanshah and Kurdistan Provinces, western Iran (Moosa-Kazemi et al. 2015, Macan 1950), Zarrin-Shahr and Mobarakeh areas of Isfahan Province, center of Iran (Mousa-Kazemi et al. 2000a), Ardabil Province, northwestern Iran (Yaghoobi-Ershadi et al. 2001), Rasht County of Guilan Province, northern Iran (Azari-Hamidian et al. 2002b) and in Iranshahr, southeastern part of the country (Ghanbari et al. 2005). Three genotypes named X, Y, and Z within *An. superpictus* during the molecular study were reported in Iran (Oshaghi et al. 2008). By now, there are no reports about the genotypes of this species in Golestan Province. However, it needs to be studied in the future.

In our study, *An. hyrcanus* was found as the dominant species in larval habitats followed by *An. maculipennis* in Kalaleh County. *An. hyrcanus* larvae were collected from habitats with varieties of 95.5% permanence, 88% stagnant water, and were only collected in clear water, natural habitats without vegetation. Different sunlight situations characterized the larval habitats of this species. The current species prefer the permanent habitats to temporary larval habitats. Moreover, *An. pseudopictus* prefers the habitats with slow running water while *An. hyrcanus* prefers the stagnant water habitats.

Three species of the hyrcanus Group including *An. hyrcanus*, *An. peditaeniatus* and *An. pseudopictus* have been reported in Iran (Azari-Hamidian et al. 2006, Azari-Hamidian 2007a). Several species of *An.hyrcanus* Group have been reported as the malaria vectors in the Oriental and Palearctic Regions, however, this species was reported as a potential malaria vector based on the molecular study in Guilan Province (Djadid et al. 2009). Shahgudian (1960) made mention of *An. nigerrimus* species as a variety of *An. hyrcanus* in its systematic key, Moreover, identification of these species is very difficult and is based on this systematic key and this old record needs to be verified. Glick (1992) published keys for the identification of female anophelines of southwest Asia, which mentioned the females of the Hyrcanus Group, however these characters were not reliable for distinguishing the females of *An. peditaeniatus* from other species of hyrcanus group. One character distinguished the larvae of *An. hyrcanus* from those of *An. pseudopictus* (Darsie and Samanidou-Voyadjoglou 1997). *An. hyrcanus* and *An. pseudopictus* were reported as a single species in southeastern France based on PCR technique (Ponçon et al. 2008). ‘The systematics of the Iranian species of the Hyrcanus Group’ was published by Azari-Hamidian and Harbach in 2009.

In our study, *Cx. pipiens* larvae were identified in this area based on the larval seta 1 of abdominal segments III and IV. This character was observed as double seta in all of *Cx. pipiens* larvae samples and this confirmed the occurrence of *Cx. pipiens* species. This species is cosmopolite and is distributed in all parts of the country (Zaim 1987, Nikookar et al. 2015).

*Culex pipiens* was predominant in larval (27.6%) and adult (28.9%) stages. Further support to this result also came from previous study, *Cx. pipiens* was reported as the dominant species in Yazd Province (Dehghan et al. 2010). *Culex pipiens* was reported as the predominant species in Guilan Province and dominant species in Isfahan Province (Mousa-Kazemi et al. 2000a, Azari-Hamidian 2007b). Larval habitats of this species were diverse in Kalaleh County but all samples of this species were collected in natural habitats. Similarly, Zaim (1987) cited the fresh water environments such as marshes, channels and artificial irrigation and rain-filled pools and drums as the main larval habitats of *Cx. pipiens.* Distribution and abundance of *Cx. pipiens* species were in close relationship with economic activities and development of new territories (Vinogradova 2000). Underground train systems, coal mines, drains, wells, septic tanks, abandoned and variety of the natural and artificial habitats were reported as the main larval habitats of *Cx*. *pipiens* (Horsfall 1955, Zaim 1987, Harbach 1988).

In our study, *Cx. pipiens* larvae were collected only in natural larval habitats, further support of this result came from the previous study, Dow (1953), Lotfi (1970, 1976), Yaghoobi-Ershadi et al. (1986), Zaim (1987) and Azari-Hamidian (2007b) found this species mostly in natural habitats. Moreover, Mousa-Kazemi et al. (2000), Azari-Hamidian et al. (2002b) have reported the presence of these species from rice fields and man-made habitats respectively. Water and sewage wells as well as house ponds were reported as the main habitats of *Cx. pipiens* in cities (Golestani 1967, Lotfi 1976, Zaim 1987, Dehghan et al. 2010, 2011).

In our study, *Cx. theileri* was found as predominant species at larvae and adult stages. This species was known as one of the predominant species in Northwest of Iran (Azari-Hamidian et al. 2009). Moosa-Kazemi et al. (2010) had reported that the *Cx. theileri* species was the second dominant species in Kurdistan Province followed by *Cx. pipiens*. Larval habitats of this species in our research were found as natural habitats, and permanent habitats with vegetation such as irrigation ditches, different pools, open cisterns, dis-used wells seepage water and swamps (Harbach 1988).

Lotfi (1970, 1976) had reported that the predominant species were in larval habitats such as grassy and ponds, rice fields, seepages and agricultural pools in Iran.

In our study, *Cx. theileri* larvae was collected from different types of habitats with 73% permanence, 64% slow running water, turbidity of 64% and 87% in natural habitats. The breeding place preferences of this species were full and partial sunlight habitats. In parallel, Azari-Hamian (2007b) had reported different types of larval habitats. Dehghan et al. (2010) reported that the larval habitats of this species were swam plants, permanent, and with vegetation outside or inside water environments in Hamadan Province. Dow (1953) reported that the larvae of *Cx. theileri* were accumulated in the pit and irrigation channels and water intakes and shallow rivers and river beds. Larval habitats of the species have been found as algae, water intake and water pits, and a crock pot, household pits along the river margins, floating and submerged plants (Horsfall 1955). Natural and artificial habitats of *Cx theileri* were cited in the Country (Dow 1953, Yaghoobi-Ershadi et al. 1986). Mousa-Kazemi et al. (2000) also discovered the presence of *Cx theileri* larvae in the rice field, however Zaim (1987) had reported their presence in natural habitats. Natural habitats such as pools were reported as the main habitats of this species (Azari-Hamidian et al. 2002b, 2007b).

In this study, more samples of *Cx. hortensis* larvae were collected from natural habitats. Moreover, this species prefers permanent and stagnant water habitats. This species is associated with *An. superpictus* and *Oc. Geniculatus*, both of which prefer habitats without vegetation so that 66% of the larval samples of this species were collected in habitats without vegetation. *Culex hortensis* species were found more in muddy beds and habitats with full and partial sunlight.

Horsfall (1955) had reported that the main larval habitats of *Cx. hortensis* were algal mats, seeps, brackish pools, domestic containers, and cement channel. This species was reported in pools in the river beds, the irrigation ditches, small, spring pools of the river banks and shallow pools (Dow 1953). Natural habitat was reported as the main habitat of this species (Zaim 1987, Azari-Hamidian 2007b). This species was collected in seepages and agricultural water storage pools (Lotfi 1976).

In our study, *Cx. perexiguus* was collected and reported at the first timein Golestan Province. More of these species were collected in natural habitats. Mousa-Kazemi et al. (2000a) have reported the occurrences of *Cx. pipiens*, *Cx. theileri* and *Cx. perexiguus* in Zarrin-Shahr and Mobarakeh areas of Isfahan Province.

By now, 5 species of *Culiseta* have been reported in Iran and they included: *Cs. Allotheobaldialongiareolata*, *Cs. Culisetaalaskaensis*, *Cs. Culiseta) annulata*, *Cs.* (*Culicella*) *morsitans* and *Cs.* (*Culiseta*) *subochrea* (Azari-Hamidian 2005). In our studied species, *Cs. logiareolata* and *Cs. subochrea* were collected. There is little information available about the ecology of the *Culiseta* species. Larval habitats of *Cs. longiareolata* contained organic materials with high abundance in artificial pits of Yazd Province (Dehghan et al. 2010). Association of this species with *Cx. pipiens* was found in the larval habitats which were contaminated with soil and wastewater in drinking troughs made of cement, a place to store water for animals and livestock in Yazd Province (Dehghan et al. 2010). In our study, *Cs. longiareolata* larvae were abundant, followed by *Cx. pipiens* and more were collected from permanent, stagnant and full sunlight habitats with vegetation. In contrast, the larval habitat of this species was without vegetation in Hamadan Province (Dehghan et al. 2011). This species has high adaptability to different ecological conditions. More *Cs. subochera* were collected in natural and temporal habitats. Similarly, these species were collected in the same larval habitats (Zaim 1987) while all the other larvae of the species were collected from permanent habitats in Hamadan Province. Moreover, *Cs. subochrea* had low abundance in this area. This species has been identified in various studies in Iran. Similarly, this species was collected as the lowest species in Hamedan Province (in terms of abundance), located in the western part of Iran. They prefer the habitats with turbid water to clear water and full sunlight habitats to shaded habitats. In Hamadan area of western Iran, the larval habitat of this species was reported as the same larval habitat in our study in turbid to clear water and full sunlight habitats. Unlike the previous study, we found more larvae in shaded habitats (Dehghan et al. 2011).

In our study, 3 species of *Ochlerotatus* including *Oc. caspius*, *Oc. echinus* and *Oc. geniculatus* were collected; Nikookar et al. (2015) had reported the occurrence of *Oc. echinus* and *Oc. geniculatus* in tree hole habitats in northern part of Iran. In our study, all the current three *Ochlerotatus* species preferred the permanent habitats with slow-running water and muddy bed. In addition, *Oc. echinus* preferred the clear water habitats while *Oc. caspius* preferred the larval habitats with turbid water and *Oc. geniculatus* was found in habitats without vegetation.

*Ochlerotatuscaspius* was reported as a potent vector for Rift Valley fever viruses as well as *Dirofilaria immitis* in the world (Azari-Hamidian 2006). This species loves feeding more on mammals and human and was found more in their dwellings (Azari-Hamidian 2006). In our research, this species comprised 7.8% of the larval collection and 9% of adult catches by animal baited trap collection method in various areas of Golestan Province, northeastern Iran. Further support to this result comes from some previous studies carried out in Kermanshah and Kurdistan Provinces, western Iran (Mousa-Kazemi et al. 2015), Zarrin-Shahr and Mobarakeh areas of Isfahan Province, center of Iran (Mousa-Kazemi et al. 2000a), Guilan Province, northwestern Iran (Azari-Hamidian et al. 2002a), Bushehr Province, southern Iran (Dow 1953), Eastern part of the country (Minar 1974) and various parts of Iran (Zaim 1987). In Kurdistan Province, *Cx. theileri* was next in abundance after this species (Moosa-Kazemi et al. 2010).

*Ochlerotatusechinus* was distributed in the Mediterranean region, north of Africa and southern Europe. In our study, out of the 9% total larvae collected from adult catches using animal baited traps in various areas of Golestan Province-northern Iran, 7.8% were *Oc. echinus.* In parallel, this species was reported in Mazandaran Province (Zaim 1987, Nikookar et al. 2015). This species has been reported in Guilan Province, northern Iran (Azari-Hamidian et al. 2002a).

*Ochlerotatusgeniculatus* was distributed in the Palearectic Region, Europe, North of Africa and Southeast Asia. In our research, this species comprised 1.5% of the larval collection and 3.9% of adult catches by animal baited traps in various areas of Golestan Province, northern Iran. This species was reported for the first time in Mazandaran Province, northern Iran (Gutsevich 1943). This species has been reported in Guilan Province, northern Iran (Azari-Hamidian et al. 2002a).

## Conclusion

The present investigation indicates some biological characteristics of mosquitoes in the northern areas of Iran. Because of diversity in larval habitats and variety in species of mosquito in the County, results of this study could be useful in vector control programs. Several species of *Anopheles* were found in a lot of areas in the county. The larval habitats of *Anopheles* were found and reported in permanent habitats with clear water. Besides, the larvae of *An. superpictus* and *An. maculipennis* species which are the main vectors of malaria in the north of Iran were reported in habitats with vegetation, under full and partial sunlight situations and muddy and sandy substrates that are important in larviciding programs. Bionomic studies of other mosquitoes need to be more rigorously studied in the future. Also, more studies should be obtained in order to complete information about of bionomics of mosquitoes in other parts of Iran.
